# No association between fiber intake and prostate cancer risk: a meta-analysis of epidemiological studies

**DOI:** 10.1186/s12957-015-0681-8

**Published:** 2015-08-28

**Authors:** Tao Sheng, Rui-lin Shen, Huan Shao, Tian-hong Ma

**Affiliations:** Department of Urology, Jiaxing Affilated Hospital of Zhejiang Chinese Medical University, Jiaxing, Zhejiang Province China; Department of Pharmacy, Jiaxing Affilated Hospital of Zhejiang Chinese Medical University, Zhongshan East Road 1501, Jiaxing, Zhejiang Province 314001 China

**Keywords:** Prostatic neoplasms, Dietary fiber, Meta-analysis, Epidemiology

## Abstract

**Background:**

The findings of epidemiologic studies on the association between fiber intake and prostate cancer risk remain conflicting. We aimed to examine this association by conducting a meta-analysis of epidemiological studies.

**Methods:**

Relevant studies were identified by PubMed (1966 to March 2015) and Embase (1974 to March 2015) database search through March 2015. We included epidemiological studies that reported relative risks (RRs) or odds ratios (ORs) with 95 % confidence intervals (CIs) for the association between dietary fiber intake and prostate cancer risk. Random effects models were used to calculate the summary risk estimates.

**Results:**

For the highest compared with the lowest dietary fiber intake, a significantly decreased risk with prostate cancer was observed in case-control studies (OR = 0.82; 95 % CI, 0.68–0.96), but not in cohort studies (RR = 0.94; 95 % CI, 0.77–1.11). The combined risk estimate of all studies was 0.89 (95 % CI, 0.77, 1.01). A significant heterogeneity was observed across studies (*p* = 0.005). There was no evidence of significant publication bias based on Begg’s funnel plot (*p* = 0.753) or Egger’s test (*p* = 0.946).

**Conclusions:**

This meta-analysis suggests the absence of evidence for association between dietary fiber intake and prostate cancer risk.

## Background

Prostate cancer is the second most common cancer among men in the world, with 1.1 million new cases diagnosed in 2012 worldwide, accounting for about 7.9 % of all cases of cancer [1]. The high prevalence and incidence of prostate cancer have resulted in a large public health burden. Age and family history are well-established and strong risk factors for prostate cancer [2]. Environmental factors such as diet are believed to play an important role in the prevention of prostate cancer because of the wide international variation in incidence [3].

Although dietary factors have long been suspected to be implicated in the development of prostate cancer, no major modifiable risk factor has been established. During the last few years, increased intake of dietary fibers has been associated with decreased risk of several cancers, such as colorectal, breast, ovarian, and upper aerodigestive tract cancers [4–7]. However, results from epidemiological studies regarding prostate cancer are sparse and inconsistent. The 2007 World Cancer Research Fund (WCRF) Second Expert Report concluded that the data were too inconsistent to draw a conclusion on the association between dietary fiber intake and prostate cancer risk [8]. Since that report was released, five prospective studies have been published on this association [9–13]. To quantitatively assess the accumulated evidence for a role of dietary fiber consumption on prostate cancer risk, we carried out a systematic review and meta-analysis of published epidemiological studies.

## Methods

### Selection of studies

Two authors performed a computerized blinded search of MEDLINE (1966 to March 2015) and Embase (1974 to March 2015) databases for relevant epidemiologic studies of dietary fiber consumption in relation to the risk of prostate cancer published in English. Additional publications identified by hand-searching of references of retrieved articles were also included. For computer searches, we used the following words in any field: “fiber” or “fibre” combined with “prostate carcinoma” or “prostatic cancer” or “prostate cancer” or “prostatic carcinoma”. Studies were included in the meta-analyses if they presented estimates of the odds ratio (OR) or relative risk (RR) and the corresponding confidence interval (CI) from a case-control or cohort study on the association between fiber intake and incidence of prostate cancer. When multiple reports were published on the same study population, we included the study with the largest number of cases.

**Fig. 1:**
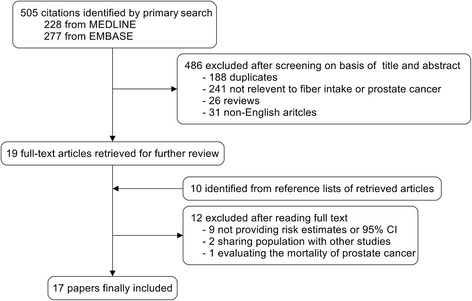
Flowchart of study selection

Figure 1 gives the flowchart for selection of articles. The primary literature search identified 505 records. After screening the titles and abstracts, 486 articles were excluded because they were either duplicates, review articles, or irrelevant to the current study. Nineteen full-text papers were retrieved. In addition, we included ten studies after reviewing reference lists of retrieved articles or preceding reviews. Twelve studies [14–25] were excluded mostly because of insufficient information to compute its RR or OR and 95 % CI. Finally, we identified 5 prospective studies [9–13] and 12 case-control studies [26–37] with data that were eligible for inclusion in the meta-analysis.

### Data extraction and classification

The following pieces of information were extracted from published studies: the name of the first author, the year of publication, the country in which the study was conducted, study design, year of follow-up (cohort studies), year of data collection (case-control studies), sample size, evaluation of exposures, the RR or OR and its 95 % CIs, exposure assessment and range of exposure, and adjusted covariates. Data extraction was conducted independently by two authors, with disagreements resolved by consensus. Considering that prostate cancer is a relatively rare disease, the RR was assumed approximately the same as OR, and the OR was used as the study outcome. If a study provided several ORs, we extracted the ORs reflecting the greatest degree of control for potential confounders. Oishi et al. [26] presented two ORs for benign prostatic hyperplasia (BPH) and hospital controls, respectively. We chose the risk estimate comparing prostate cancer with hospital controls instead of BPH because it may increase the chance of diagnosing an incidental prostate cancer [38].

### Quality assessment

The study quality was assessed using the nine-star Newcastle-Ottawa Scale (The Newcastle-Ottawa Scale for assessing the quality of nonrandomized studies in meta-analyses. Ottawa, Canada: Dept of Epidemiology and Community Medicine, University of Ottawa. http://www.ohri.ca/programs/clinical_epidemiology/oxford.htm). NOS is an eight-item instrument that allows for the assessment of the patient selection, study comparability, and exposure (for case-control study) or outcome (for cohort study). The range of possible scores is 0–9. The study with score more than 6 was considered of high quality.

### Statistical analysis

We used random effects models to calculate summary ORs and 95 % CIs for the highest vs. the lowest levels of dietary fiber because it used a combination of within-study variance and between-study variance for computing weights. We evaluated the heterogeneity among studies with the Cochrane *Q* test [39] and *I*^2^ score [40]. We also estimated the 95 % prediction interval, which further accounts for between-study heterogeneity and evaluates the uncertainty for the effect that would be expected in a new study addressing that same association [41]. To explore the sources of heterogeneity across studies, subgroup analyses were conducted according to study design, study quality, geographic region, and method of dietary assessment. Because adjustments for confounding factors were not consistent between the studies, we also conducted the subgroup analysis according to whether the risk estimates had been adjusted for family history of prostate cancer, body mass index (BMI), and total energy intake. In addition, we further performed a sensitivity analysis to explore sources of heterogeneity. Each study was omitted at a time to assess robustness of the results. In addition to those methods, the Galbraith plot was also used to detect the possible sources of heterogeneity, and a re-analysis was conducted with exclusion of the studies possibly causing the heterogeneity. Meta-regression was also applied to measure the subgroup interaction. The *p* value for interaction between two groups is the comparison of subgroup vs. the other. We used *p* < 0.10 as the indicator of significant interaction. Publication bias was assessed by Begg’s [42] and Egger’s [43] test. All analyses were performed by using STATA version 11.0 (StataCorp). A *p* value < 0.05 was considered significant.

## Results

The characteristics of these studies and the variables evaluated are listed in Table 1. Six studies were conducted in North America [10, 31, 33, 34, 36, 37], seven in Europe [9, 11, 12, 28, 30, 32, 35], two in Japan [13, 26], one in South Africa [27], and one in Uruguay [29]. Overall, this meta-analysis included more than 8000 cases of prostate cancer. Information on fiber intake was obtained by interview or self-administered questionnaire using food frequency questionnaires (FFQ) except one using 24-h dietary record [12]. All of the included studies adjusted for age, and 14 of them included adjustment for energy intake [6, 9–13, 28–31, 33–36], 8 adjusted for family history [6, 10, 12, 29–31, 36, 37], and 8 adjusted for BMI [10, 12, 13, 29, 30, 33, 36, 37].

**Table 1:** Study characteristics of published cohort and case-control studies on dietary fiber intake and prostate cancer

Authors and publication year	Study design	Country	Study period	Cases/subjects	Exposure range	RR (95 % CI)	Variables of adjustment	Study quality^a^	Other variables evaluated	Assessment
Oishi et al. 1988 [26]	HCC	Japan	1981–1984	100/200	Ever vs. none	0.78 (0.45–1.37)	Age	5	None	Interview FFQ (31 items)
Walker et al. 1992 [27]	PCC	South Africa	1998–1990	166/332	≥15 vs. <15 g/day	0.6 (0.4–1.0)	Age	6	None	Interview FFQ (unknown items)
Andersson et al. [35]	PCC	Sweden	1989–1994	526/1062	The highest quartile (≥25.9 g/day) vs. the lowest (<15.9 g/day)	0.82 (0.58–1.15)	Age, energy	6	Advanced prostate cancer	Interview and self-administered questionnaire FFQ (68 items)
Vlajinac et al. 1997 [28]	HCC	Serbia	1990–1994	101/303	The highest tertile vs. the lowest	4.02 (1.38–11.73)	Age, residence, energy, protein, fat total, saturated fatty acids, carbohydrate, total sugar, retinol, retinol equivalent, a-tocopherol, folic acid, vitamin B_12_, sodium, potassium, calcium, phosphorus, magnesium, and iron	6	None	Interview FFQ (150 items)
Deneo-Pellegrini et al. 1999 [29]	HCC	Uruguay	1993–1997	175/408	The highest quartile (≥27.2 g/day) vs. the lowest (<18.2 g/day)	1.5 (0.8–2.6)	Age, residence, urban/rural status, education, family history of prostate cancer, BMI, and total energy intake	6	None	Interview FFQ (64 items)
Ramon et al. 2000 [30]	HCC	Spain	1994–1998	270/704	The highest quartile (≥39.5 g/day) vs. the lowest (<13.1 g/day)	1.0 (0.7–1.5)	Age, residence, family history of prostate cancer, BMI, and energy intake	8	None	Interview FFQ (141 items)
Lu et al. 2001 [31]	PCC	USA	1993–1997	65/197	The highest quartile (≥13.7 g/day) vs. the lowest (<7.9 g/day)	1.81 (0.55–5.96)	Age, race, education, alcohol drinking, pack-years of smoking, family history of prostate cancer, and total dietary caloric intake	8	None	Interview FFQ (98 items)
Pelucchi et al. 2004 [32]	HCC	Italy	1991–2002	1294/1745	The highest quintile (≥21.1 g/day) vs. the lowest (<12.3 g/day)	0.93 (0.71–1.22)	Age, study center, education, family history of prostate cancer, smoking habit, alcohol consumption and total energy intake	7	Insoluble fiber, cellulose, vegetable fiber, fruit fiber, grain fiber.	Interview FFQ (78 items)
McCann et al. 2005 [33]	PCC	USA	1986–1991	433/971	The highest quartile (>38 g/day) vs. the lowest ≤15 g/day	1.21 (0.73–2.01)	Age, education, BMI, cigarette smoking status, and total energy	7	None	Interview FFQ (172 items)
Walker et al. 2005 [34]	HCC	Canada	1997–1999	80/414	The highest tertile vs. the lowest	1.10 (0.58–2.07)	Age, alcohol, energy, fat, carbohydrate, calcium, protein, and cholesterol intake	6	None	Interview FFQ (66 items)
Lewis et al. 2009 [36]	HCC	USA	1998–2004	478/860	The highest tertile (≥20.7 g/day) vs. the lowest (<13.7 g/day)	0.56 (0.35–0.89)	Age, education, BMI, smoking history, family history of prostate cancer in first-degree relatives, and total caloric intake	6	None	Self-administered questionnaire FFQ (100 items)
Suzuki et al. 2009 [9]	Cohort	Europe	1993–2007	2747/142,590	The highest quintile (≥30.4 g/day) vs. the lowest (<17.8 g/day)	1.02 (0.87–1.19)	Age, energy intake, height, weight, smoking, education, and marital status	8	Vegetables fiber, fruit fiber, cereal fiber Local, advanced, low-grade, and high-grade prostate cancer	
Nimptsch et al. 2011 [10]	Cohort	USA	1986–2002	5112/49,934	The highest quintile (≥26 g/day) vs. the lowest (≤15.4 g/day)	1.01 (0.92–1.12)	Age, BMI, height, history of diabetes, family history of prostate cancer, race, smoking, vigorous physical activity, energy intake, alcohol intake, calcium intake, alphalinolenic acid, and tomato sauce	7	Local, advanced, low-grade and high-grade prostate cancer	Self-administered questionnaire FFQ (131 items)
Drake et al. 2012 [11]	Cohort	Sweden	1991–2009	817/8128	The highest quintile (≥23.7 g/day) vs. (17.6 g/day) the lowest	1.15 (0.89–1.49)	Age, year of study entry, season of data collection, energy intake, height, waist, physical activity, smoking, educational level, birth in Sweden, alcohol, calcium, selenium	9	Low-risk, high-risk, and symptomatic prostate cancer	Interview FFQ (168 items)
Deschasaux et al. 2014 [12]	Cohort	France	1994–2007	139/3313	The highest quartile vs. the lowest	0.47 (0.27–0.81)	Age, energy intake without alcohol, intervention group, number of 24-h dietary records, smoking status, educational level, physical activity, height, BMI, alcohol intake, family history of prostate cancer, prostate-specific antigen, calcium intake, processed meat intake, tomato product intake, vitamin E intake, and blood selenium	7	Soluble fiber, insoluble fiber, cereal fiber, vegetable fiber, fruit fiber, legume fiber	24-h dietary record
Vidal et al. 2015 [37]	HCC	USA	2007–2012	156/430	The highest tertile vs. the lowest	0.79 (0.31–1.97)	Age, race, family history, caloric intake, carbohydrate intake, BMI, diabetes, physical activity, alcohol, and smoking status	6	Low-grade and high-grade prostate cancer	Interview FFQ (61 items)
Sawada et al. 2015 [13]	Cohort	Japan	1995–2009	825/43,435	The highest quartile vs. the lowest	1.00 (0.77, 1.29)	Age, public health center area, smoking status, drinking frequency, marital status, BMI, and intakes of green tea, genistein, SFAs, and carbohydrate	7	Soluble fiber, insoluble fiber, local and advanced prostate cancer	Self-administered questionnaire FFQ (138 items)

*PCC* population-based case-control studies, *HCC* hospital-based case-control studies, *FFQ* food-frequency questionnaire, *BMI* body mass index

^a^Evaluated by nine-star Newcastle-Ottawa Scale

**Fig. 2:**
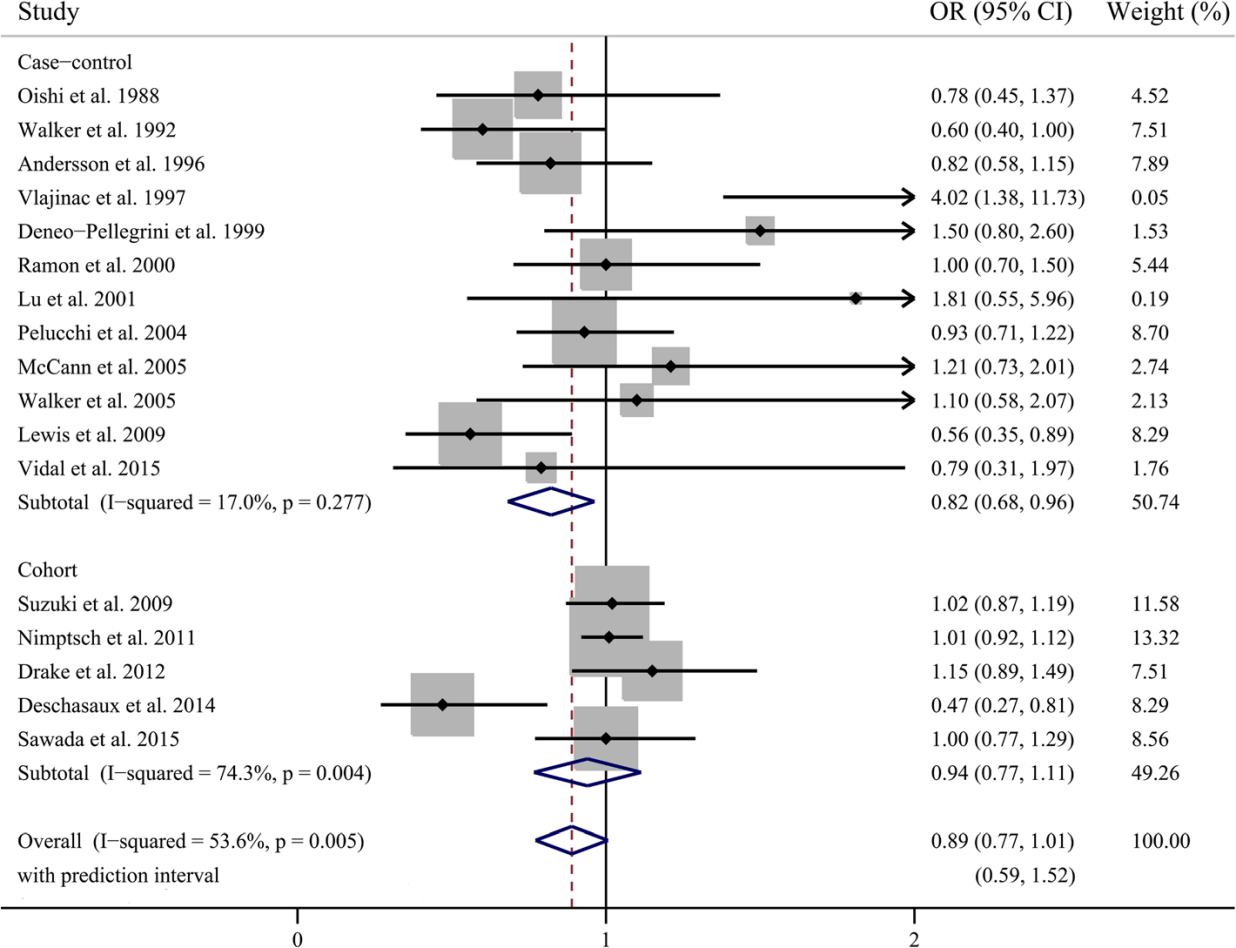
Pooled results for 12 case-control and 5 cohort studies of dietary fiber intake and prostate cancer risk

**Fig. 3:**
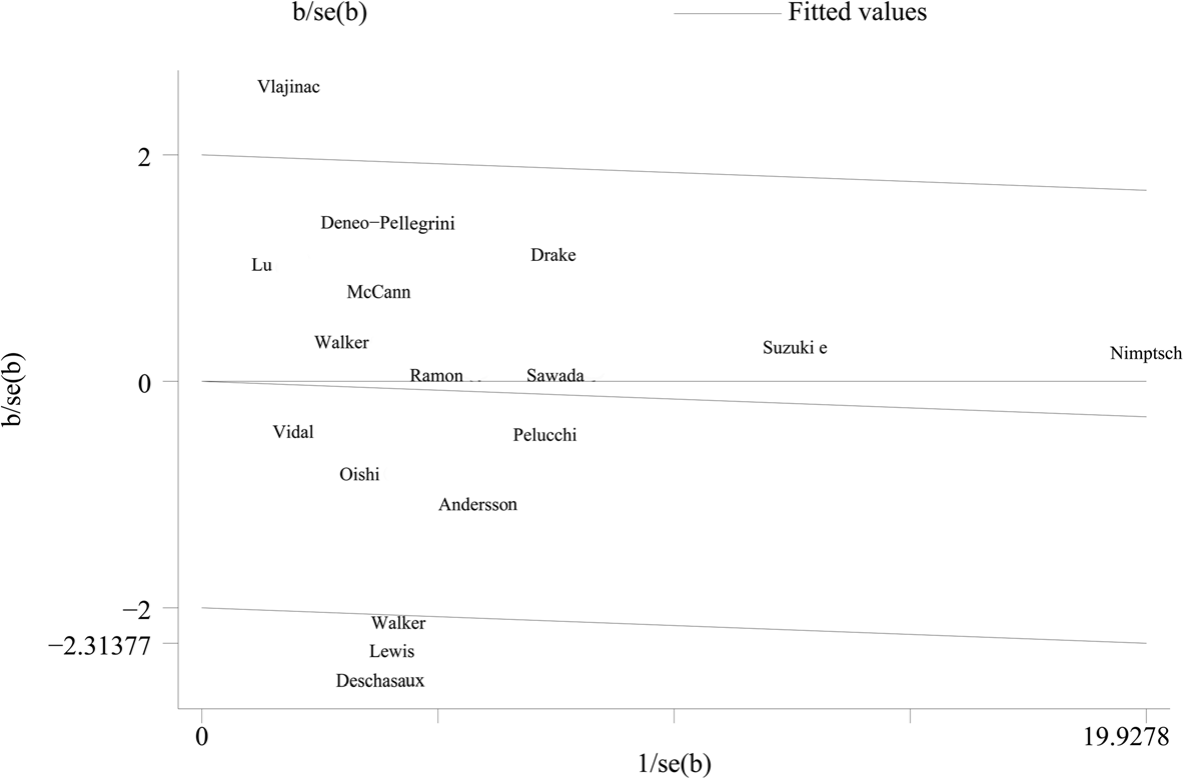
Galbraith plot analysis indicated that four studies were the potential source of heterogeneity

**Fig. 4:**
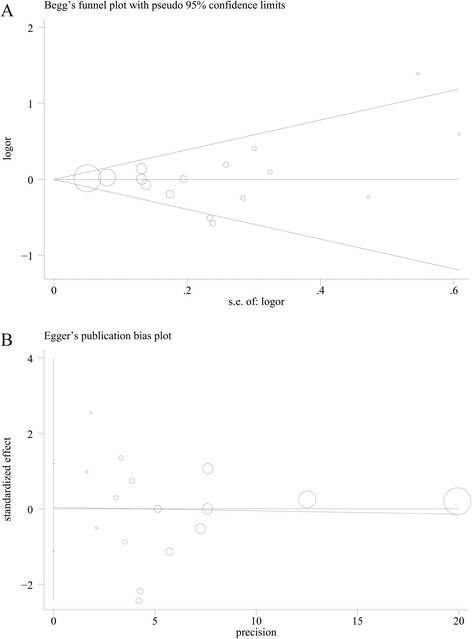
Publication bias which was estimated by Begg’s test (**a**) and Egger’s test (**b**)

As shown in Fig. 2, a statistically significant protective effect of dietary fiber intake on prostate was observed in case-control studies (OR = 0.82; 95 % CI, 0.68–0.96), while no such effect was observed in cohort studies (RR = 0.94; 95 % CI, 0.77–1.11). There was no evidence of heterogeneity among case-control (*p* = 0.277, *I*^2^ = 17 %), but significant heterogeneity among cohort studies (*p* = 0.004, *I*^2^ = 74.3 %). When all these studies were analyzed together, no association was observed between fiber intake and risk of prostate cancer (summary OR = 0.89; 95 % CI, 0.77–1.01), with significant heterogeneity among studies (*p* = 0.005, *I*^2^ = 53.6 %). The wide 95 % prediction interval also included the null value and reflected the significant heterogeneity (0.59, 1.52). In a sensitivity analysis excluding one study at a time, the summary OR for prostate cancer ranged from 0.87 (0.75 to 0.99) when the study by Drake et al. [11] was excluded to 0.93 (0.83 to 1.03) when the study by Deschasaux et al. [12] was excluded. Through the Galbraith plot, four studies were identified as the major sources of heterogeneity (Fig. 3). After excluding these four studies, there was no study heterogeneity (*p* = 0.915, *I*^2^ = 0), and the overall association turned out to be null (OR 1.00, 95 % CI 0.93–1.07). There was no evidence of significant publication bias either with the Egger’s test (*p* = 0.946) or Begg’s funnel plot (*p* = 0.753) (Fig. 4).

Next, we performed subgroup analyses by study quality, geographical region, and the method of exposure assessment (Table 2). When we stratified by study quality, more significant association was observed in studies of low-quality (OR 0.73, 95 % CI 0.56–0.90) compared with studies of high-quality (OR 0.96, 95 % CI 0.83–1.08). Considering the geographic area, the pooled OR was 0.90 (95 % CI, 0.65–1.16) in European studies, 0.90 (95 % CI 0.64–1.06) in North American studies, and 0.95 (95 % CI, 0.72–1.17) in Japanese studies. When separately analyzed by exposure assessment, the ORs were 0.93 (95 % CI 0.76–1.09) for studies that used an interview and 0.94 (0.76–1.10) for with a self-administered questionnaire, respectively.

**Table 2:** Subgroup analyses of odds ratios for the association between fiber intake and risk of prostate cancer

Outcome of interest	No. of studies	OR (95 % CI)	*p*_heterogenity_	*I* ^2^ (%)	*p* for interaction
Total dietary fiber	17	0.89 (0.77, 1.01)	0.005	53.6	
Study design					
Cohort	5	0.94 (0.77, 1.11)	0.004	74.3	0.202
Case-control	12	0.82 (0.68, 0.96)	0.277	17.0	
Study quality					
Low	8	0.73 (0.56, 0.90)	0.335	12.2	0.033
High	9	0.96 (0.83, 1.08)	0.04	51.7	
Geographical region					
Europe	7	0.90 (0.71, 1.09)	0.01	63.5	0.937
North America	6	0.90 (0.64, 1.16)	0.059	53.1	
Japan	2	0.95 (0.72, 1.17)	0.41	0	
Assessment					
Interview	11	0.94 (0.79, 1.10)	0.313	13.8	0.931
Questionnaire	4	0.93 (0.76, 1.09)	0.02	69.7	
Family history					
Yes	8	0.84 (0.62, 1.05)	0.002	69.4	0.44
No	9	0.94 (0.81, 1.08)	0.187	29.0	
BMI					
Yes	8	0.87 (0.66, 1.08)	0.001	70.3	0.695
No	9	0.92 (0.79, 1.06)	0.21	26.4	
Energy intake					
Yes	14	0.91 (0.78, 1.04)	0.007	55.1	0.507
No	3	0.81 (0.54, 1.07)	0.14	49.2	
Multiple confounders^a^					
Yes	6	0.82 (0.54, 1.09)	0.306	14.4	0.387
No	11	0.95 (0.84, 1.05)	<0.001	77.7	
Tumor stage					
Local	3	0.98 (0.89, 1.08)	0.24	30.5	0.562
Advanced	4	0.93 (0.79, 1.07)	0.24	29.3	
Source of intake					
Cereal fiber	3	1.05 (0.94, 1.16)	0.52	0	0.02
Fruit fiber	3	0.92 (0.81, 1.03)	0.55	0	
Vegetable fiber	3	0.87 (0.53, 1.21)	0.001	84.8	
Legume fiber	1	0.55 (0.32, 0.95)	NA	NA	
Solubility					
Soluble fiber	2	0.87 (0.52, 1.22)	0.13	57.2	0.777
Insoluble fiber	3	0.80 (0.46, 1.13)	0.005	81.0	

^a^Multiple confounders refer to effect estimates adjusted for at least family history, BMI, and energy intake

We also investigated the impact of some confounding factors on the estimates of ORs (Table 2). Family history is the established risk factor for prostate cancer; BMI and energy are potential confounders of the relationship between fiber intake and the risk of prostate cancer. We found that the non-significant relationships between prostate cancer and fiber intake were consistent in all subgroups, whether controlled for family history, BMI, and energy intake or not. Moreover, six studies in our analysis adjusted for these three confounders simultaneously. Therefore, we examined whether more thoroughly adjusting for potential confounders affected the pooled OR. The effect estimates for studies that adjusted for these three confounders or not were ORs of 0.82 (95 % CI 0.54–1.09) and 0.95 (0.84–1.05), respectively.

In addition, after stratification according to food source and solubility, none of the subtypes could lower the incidence of prostate cancer significantly, except for legume fiber, though it is based on only one cohort study [12]. We also pooled the ORs by clinical characteristics of prostate cancer. The summary ORs did not indicate that high fiber intake had a significant protective association with low- or high-stage disease (Table 2).

## Discussion

This is the first meta-analysis for clarification of the association between fiber intake and risk of prostate cancer. Twelve case-control studies and 5 prospective studies involving more than 8000 cases were included in our study. The results suggested no significant association between dietary fiber intake and prostate cancer incidence.

Although the pooled analysis from the case-control studies suggested a significant reduction in risk, the results from the cohort studies were non-significant, suggesting that our conclusion depend mainly on the cohort studies. It is generally thought that cohort studies provide stronger evidence regarding an association than case-control studies because they are less prone to differential recall of dietary habits or selection bias. Therefore, the evidence from case-control studies should be viewed with caution, particularly considering that the combined risk estimates from all studies suggested no association. In the subgroup analysis separated by study quality, we observed that fiber intake was associated with decreased risk of prostate cancer in low-quality studies, but no significant association in high-quality studies. This may account partly for the discrepancy between cohort and case-control studies, since all 5 cohort studies were high-quality studies published after 2009, while 8 of 12 case-control studies were low-quality ones. Moreover, the non-significant relationships were similar independent of study design, geographical region, method of dietary assessment, and adjustment for several essential confounders or not, further strengthening the stability of our findings.

We observed a significant heterogeneity among studies, which was partly explained by the fact that levels in the lowest and highest categories and the range of intake were various and quite heterogeneous across studies. In addition, accurate assessment of fiber intake is a challenge. A previous meta-analysis suggested that the different definition of dietary fiber between included studies may contribute to heterogeneity in the results [44]. However, only one study used the Englyst method for the definition of fiber [32]. Also, the extent to which confounding factors were controlled differed among studies, which may bring heterogeneity and resulted in inaccurate pooled estimates. For the two established risk factors, all studies included in this meta-analysis provided risk estimates adjusted for age, while 8 of 17 studies controlled for a family history of prostate cancer in their analyses [6, 10, 12, 29–31, 36, 37]. However, it is unlikely that a family history of prostate cancer is a strong confounder because it is not strongly related to fiber intake. In the subgroup analysis, results that did and did not adjust for family history did not differ in the meta-regression. The summary OR represents the combination of different types of fiber, such as soluble and insoluble fiber, and fiber from different food sources, which may have different effects on prostate cancer, though the pooled estimates of subtypes suggested no association, except for the legume fiber. Intakes of different types of fiber vary across countries, thus providing another explanation for the heterogeneity across studies. We also performed Galbraith plot analysis and identified four studies reporting extreme ORs as the potential sources of heterogeneity [12, 27, 28, 36]. No heterogeneity existed after excluding these four studies.

It has been suggested that dietary fiber may reduce prostate cancer risk possibly by increasing circulating levels of sex hormone-binding globulin [45] and improving insulin sensitivity [46]. Fiber may reduce insulin resistance through a decrease in carbohydrate absorption rate [47]. Insulin resistance and hyperinsulinemia, by decreasing insulin-like growth factor (IGF) binding proteins and increasing IGF concentrations, may stimulate prostate carcinogenesis [46]. Foods rich in dietary fiber also contain dietary lignans, which are postulated to be associated with a decreased risk of prostate cancer [48]. New evidence showed that inositol hexaphosphate (IP6), a major component of high-fiber diet, could control the progression of prostate cancer in mice due to its anti-angiogenic effects [49]. The inconsistency between experimental and epidemiology studies may be partly explained by the low bioavailability of these active ingredients in human plasma, and the drug accumulation could not achieve high levels in prostate. It was noted that most of the included studies were conducted in western countries, and the western diet is typically described as being high fat and low fiber compared with Asian diet [50], probably leading to relatively low blood levels of active ingredients in the subjects, thus the non-significant findings in the meta-analysis.

Our study has several important limitations. First, fiber intake was generally not the main focus of the included studies. Although analysis of total fiber on prostate cancer incidence was based on many studies, results for fiber subtypes and secondary outcomes of local and advanced stage disease were limited. As such, the pooled estimates were more susceptible to the influence from individual studies and should be interpreted with caution. Second, we were unable to conduct dose-responses because some studies did not provide the value of fiber intake in each category, and the number of cases and noncases by stratum were often missing in studies. Third, although most studies included in our analysis had performed adjustment for a wide range of confounders, we could not rule out the possibility that other unidentified or unmeasured factors could affect the association. Fourth, we did not sought to include unpublished data or papers in other languages, yet little evidence of publication bias was observed.

## Conclusions

In conclusion, this meta-analysis of epidemiological studies provides evidence that diets with high intake of plant-based foods rich in fiber may have no impact in the prevention of prostate cancer. Additional studies, especially large prospective cohort studies, are warranted to confirm these findings and address the effects of different fiber subtypes and secondary outcomes.

## Abbreviations

BMI: body mass index
CI: confidence interval
FFQ: Food frequency questionnaire
IGF: insulin-like growth factor
OR: odds ratio
RR: relative risk
